# Folate deficiency disturbs hsa‐let‐7 g level through methylation regulation in neural tube defects

**DOI:** 10.1111/jcmm.13228

**Published:** 2017-06-19

**Authors:** Li Wang, Shaofang Shangguan, Yu Xin, Shaoyan Chang, Zhen Wang, Xiaolin Lu, Lihua Wu, Bo Niu, Ting Zhang

**Affiliations:** ^1^ Beijing Municipal Key Laboratory of Child Development and Nutriomics Capital Institute of Pediatrics Beijing China

**Keywords:** neural tube defects (NTDs), folic acid, hsa‐let‐7 g, DNA methylation, cell migration and proliferation

## Abstract

Folic acid deficiency during pregnancy is believed to be a high‐risk factor for neural tube defects (NTDs). Disturbed epigenetic modifications, including miRNA regulation, have been linked to the pathogenesis of NTDs in those with folate deficiency. However, the mechanism by which folic acid‐regulated miRNA influences this pathogenesis remains unclear. It is believed that DNA methylation is associated with dysregulated miRNA expression. To clarify this issue, here we measured the methylation changes of 22 miRNAs in 57 human NTD cases to explore whether such changes are involved in miRNA regulation in NTD cases through folate metabolism. In total, eight of the 22 miRNAs tested reduced their methylation modifications in NTD cases, which provide direct evidence of the roles of interactions between DNA methylation and miRNA level in these defects. Among the findings, there was a significant association between folic acid concentration and hsa‐let‐7 g methylation level in NTD cases. Hypomethylation of hsa‐let‐7 g increased its own expression level in both NTD cases and cell models, which indicated that hsa‐let‐7 g methylation directly regulates its own expression. Overexpression of hsa‐let‐7 g, along with its target genes, disturbed the migration and proliferation of SK‐N‐SH cells, implying that hsa‐let‐7 g plays important roles in the prevention of NTDs by folic acid. In summary, our data suggest a relationship between aberrant methylation of hsa‐let‐7 g and disturbed folate metabolism in NTDs, implying that improvements in nutrition during early pregnancy may prevent such defects, possibly *via* the donation of methyl groups for miRNAs.

## Introduction

NTDs are thought to be caused by a combination of genetic defects and environmental factors. However, given their complexity, it is difficult to identify one specific factor or gene to explain their pathogenesis. Recent studies have shown that mouse models with the knockdown or knockout of one or a series of genes often die in utero or shortly after birth; in addition, there are few mutant models of NTDs 1, 2, 3, 4, 5, 6, 7, 8, 9, 10, 11. This background suggests that modified gene expression and not the complete loss of gene expression may be a driving force behind NTDs 12. An increasing number of studies have also implied that epigenetic modifications contribute to NTDs, but the mechanism involved in this has remained unclear.

Epidemiological studies have indicated that lower concentrations of folate, which in turn disturb one‐carbon metabolism, during pregnancy are related to an increased risk of NTDs 13, 14, 15. Previous studies by our group showed that disturbed epigenetic modifications in the context of folic acid deficiency are involved in the pathogenesis of NTDs, including DNA methylation, histone methylation and microRNA (miRNA) regulation 16, 17, 18. Intracellular folic acid is a key factor acting as a source of adequate methyl groups for the methylation of DNA and proteins, which explains why folic acid deficiency leads to changes in cytosine methylation and histone methylation that could induce NTDs. However, the mechanism by which folic acid regulates miRNAs involved in the pathogenesis of NTDs has remained unclear.

miRNA is a small non‐coding RNA molecule (containing about 22 nucleotides) that functions in RNA silencing and post‐transcriptional regulation of gene expression. It is known that DNA methylation, histone modifications and expression patterns of non‐coding RNAs are the main types of epigenetic regulation, which contribute to the modulation of chromatin structure and participate in major molecular processes in cells. Emerging evidence now supports the idea that different epigenetic modifications are not independent of each other; in fact, histone modification was found to be important for the location of DNA methylation 19, 20, 21, and some studies have provided evidence that DNA methylation is essentially involved in dysregulated miRNA expression 22, 23, 24, 25. Interestingly, at least half of the promoter regions for miRNAs are predicted to be in close proximity to CpG islands and their methylation frequency is predicted to be at least one order of magnitude higher than that of protein‐coding genes 12. New studies in human cell lines have also shown that folic acid deficiency and DNA hypomethylation can lead to the misexpression of miRNAs 26, 27, 28, 29. Against this background, a more thorough understanding of how folate deficiency contributes to the misexpression of miRNAs and their roles in NTDs should help us to clarify the mechanism behind miRNA regulation in NTDs.

In this study, 22 miRNA were selected for methylation test according to findings in cancer research, which implying a potential connection of methylation regulation on miRNAs in the pathology of cancer. Herein, changes in methylation of 22 miRNAs were explored in human cases with spinal bifida. Specifically, we measured and compared the methylation levels of hsa‐let‐7 g in NTD and control samples, along with determining the relationship between methylation modification and miRNA expression. We also investigated folic acid concentration in NTD cases to explore the association between folic acid and hsa‐let‐7 g methylation levels in cases of NTDs. In addition, we studied the biological functions of hsa‐let‐7 g to understand its roles in the pathogenesis of NTDs.

## Materials and methods

### Sample collection

All samples were obtained from a long‐term surveillance program concerning the high prevalence of NTDs in the Lüliang Mountain area of northern China. Foetuses affected with NTDs were obtained from stillbirth deliveries and medical abortions based on a diagnosis using B‐ultrasound. All case samples enrolled in this study for methylation analysis are spinal bifida. Definitive diagnosis was made by post‐mortem according to the International Classification of Disease, Tenth Revision, code Q05 spina bifida. Control samples were aborted for non‐medical reasons and were confirmed to have no deformities by post‐mortem. None of the pregnant women had received periconceptional folic acid supplementation.

A total of 107 samples were collected from July 2005 to August 2010 for this study, including 50 controls and 57 cases with spinal bifida, mixture of brain tissue and residue of brain tissue were harvested for DNA extraction.

Aborted foetuses for RNA assay were collected within 30 min. after the abortion operation, anatomy was conducted by the certified pathologists from the Capital Institute of Pediatrics, and the isolated samples were snap‐frozen in the liquid nitrogen and transferred to the study laboratories for storage at −80°C.

Blood samples from pregnant women were collected prior to abortion. Venous blood (2 ml) was collected in Vacutainer^®^ tubes (BD, Suzhou, China) without anticoagulant. Samples were immediately centrifuged at 1000g for 10 min. and separated plasma was aliquoted without reducing agents and stored.

All participants provided their informed consent, and the study protocol was reviewed and approved by the Institutional Review Board of the Capital Institute of Pediatrics in Beijing, China. Detailed clinical and socio‐economic information was collected by local doctors.

### Cell culture

The human colorectal cancer cell line HCT‐15 was obtained from the American Type Culture Collection (Manassas, VA, USA). Cells were cultured at 37°C in a humidified 5% CO_2_ atmosphere in RPMI 1640 medium (Invitrogen, Carlsbad, CA, USA) supplemented with 10% foetal bovine serum (Gibco, Gaithersburg, MD, USA). Cells in the exponential growth phase were used for subsequent experiments.

For demethylation studies, cultured cells were incubated for 72 hrs in 0, 5, 15 or 50 μM/l 5‐azacytidine (5‐Aza; Sigma‐Aldrich, Shanghai, China), a methylation inhibitor, and the medium was changed daily.

### Biochemical analyses

Folic acid in brains was measured using a competitive receptor binding immunoassay (Chemiluminescent Immunoenzyme Assay Access Immunoassay System II; Beckman Coulter, Krefeld, Germany). The intra‐assay CV for folate was 3.8–6.5%.

### DNA extraction

Genomic DNA was extracted from cells and foetal brain in cases affected by NTDs using the Maxwell^®^ 16 Tissue DNA Purification Kit (Promega, Madison, WI, USA), in accordance with the manufacturer's instructions.

### Bisulphite treatment

A total of 500 ng of DNA from each sample was bisulphite‐converted using a Methylamp DNA Modification Kit (EpiGentek, Farmingdale, NY, USA), in accordance with the manufacturer's instructions.

### EpiTect Methyl II Signature PCR Array for 22 genes

A total of 1 μg of genomic DNA from five NTD cases and five controls was used for restriction digestion using the EpiTect Methyl II DNA Restriction Kit (cat. no. 335452); relative fractions of methylated and unmethylated promoter DNA of 22 miRNAs were determined by comparing the amount in each digest with that of a mock (no enzymes added) digest using the ΔCT method, in accordance with the protocol.

### hsa‐let‐7 g methylation analyses

**Figure 1:**
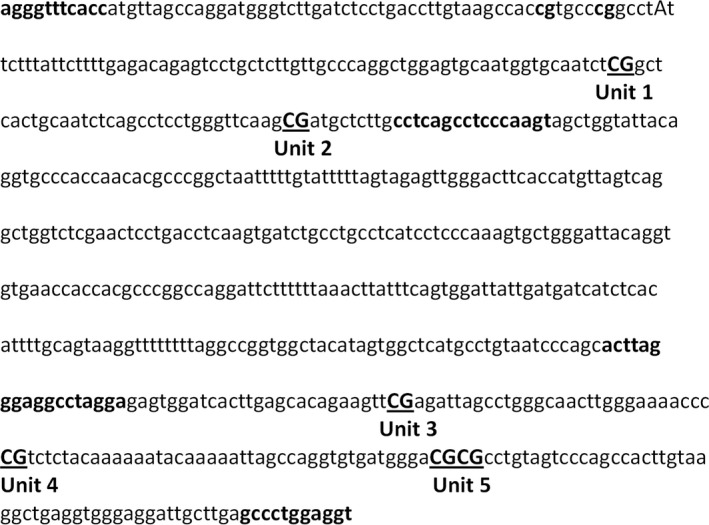
Schematic diagram of the promoter region of hsa‐let‐7 g. The sequence shown represents a fragment of 657 base pairs: chr3: 52302917–52303573. Numbers 1–5 refer to locations of the CpG unit within the fragment tested.

The Sequenom MassARRAY platform (CapitalBio, Beijing, China) was used to perform quantitative analysis of the methylation of hsa‐let‐7 g. This system uses matrix‐assisted laser desorption/ionization time‐of‐flight (MALDI‐TOF) mass spectrometry in combination with RNA base‐specific cleavage (MassCLEAVE). The detected pattern is then analysed for its methylation status. PCR primers were designed with Methprimer (http://epidesigner.com). For each reverse primer, an additional T7 promoter tag for in vivo transcription was added, as well as a 10‐mer tag on the forward primer to adjust for melting temperature differences. Two pairs of primers were used to amplify the promoter region of hsa‐let‐7 g: L1: aggaagagagATTTGGGAGGTTGAGGTAAGAGTAT; R1: cagtaatacgactcactatagggagaaggctCAAAATTTCACCATATTAACCAAAA; L2: aggaagagagTATTTAGGGAGGTTTAGGAGAGTGG; and R2: cagtaatacgactcactatagggagaaggctACCTCCAAAACTCAAACAATCCT. The location of primers is shown in Figure 1. In total, six CpG sites (which were divided into five CpG units) were examined in this region (shown in Fig. 1). The spectral methylation ratios were generated using Epityper software version 1.0 (Sequenom, San Diego, CA, USA).

### Gene expression analysis

Total RNA was extracted using the RNeasy^®^ Mini Kit (QIAGEN, Hilden, Germany). For RT‐PCR, 1 μg of total RNA was reverse‐transcribed into cDNA using the Protoscript^®^ First Strand cDNA Synthesis Kit (NEB, Ipswich, MA, USA), in accordance with the manufacturer's instructions. Real‐time PCR was performed with a 7500 Fast Real‐Time PCR System (Applied Biosystems, Beijing, China). Samples for PCR were run in triplicate. Relative mRNA levels were compared using the 2^−*ΔΔ*CT^ method, with *Gapdh* as a control. For primer sequences, see Table S1.

### Determination of cell growth using MTT assay

The MTT Cell Proliferation and Cytotoxicity Assay Kit (C0009, China) was used to detect the proliferation rate of cells. SK‐N‐SH cells were transfected with hsa‐let‐7 g or a negative control, and cell transfection was performed with Lipofectamine™ 2000, in accordance with the manufacturer's instructions. After transfection for 24 hrs, cells were trypsinized and seeded in 96‐well plates at a density of 4 × 10^3^ cells/well in 200 μl of complete medium. A total of 20 μl of 5 mg/ml MTT was put into each well at 0, 24, 48 and 72 hrs and incubated at 37°C for 4 hrs, and then the medium was replaced by 150 μl of DMSO (Sigma‐Aldrich) and shaken at room temperature for 10 min. The absorbance was measured at 570 nm.

### Cell migration analysis

SK‐N‐SH cells were trypsinized and seeded at a density of 5 × 10 ^5^ cells/well in a 24‐well chamber; hsa‐let‐7 g and a negative control were transected at a concentration of 50 μM/l. Each treatment was repeated in triplicate. After incubation at 37°C overnight, cells were looted and removed with a toothpick. Then, the cells were incubated with serum‐free medium and photographed under a microscope every 8 hrs. The obtained data were analysed using ImageJ software.

### Expression microarray analysis

Two NTD samples and three controls were included to perform genomic gene expression array. Specifically, total RNA was isolated individually from NTD‐affected and control foetuses using TRIzol reagent (Invitrogen), following the manufacturer's instructions. Concentration and quality of the isolated RNA were initially assessed by NanoDrop^®^ND‐1000 spectrophotometer and gel electrophoresis, respectively. Only RNAs that reached the quality standard of Illumina BeadArray applications were selected for the subsequent experiments. The microarray of the HumanWG‐6 v3 Expression BeadChip (Illumina, San Diego, CA, USA), which has over 47,000 probes for assessing mRNA expression, was selected to generate whole‐genome mRNA expression profiles for both NTD‐affected and normal tissue samples. DEGs were identified using the RankProd program 30, which uses a nonparametric statistical method to estimate significance levels of genes' differential expression (rank) based on a gene permutation model. For the multitest correction, we used the Benjamini–Hochberg false discovery rates (FDRs), instead of *P*‐values, to determine the DEGs with a cut‐off value of FDR <0.05.

### Statistical analyses

Data were stored in the EPI 3.1 Database (EpiData Association, Odense, Denmark) and analysed with the SPSS‐11.5 software package (McGraw‐Hill Inc., New York, NY, USA). All *P*‐values were two‐sided, and *P* < 0.05 was considered to be significant. Independent *t*‐tests were performed to evaluate the significance of any differences between test and control groups. Correlation analysis was performed with bivariate correlations. Odds ratios (ORs), which were used to evaluate the incidence of NTDs in relation to methylation levels, were calculated with the 95% CI. The adjusted ORs (AORs) were calculated by logistic regression. miRNA target genes were predicted using miRWALK.

## Results

Samples from a total of 57 spinal bifida cases were obtained for methylation analysis, 50 non‐NTD control foetuses, aborted for non‐medical reasons, were obtained from the same geographical region and matched according to gender and gestational weeks. The characteristics of these subjects are shown in Table 1.

**Table 1:** Characteristics of the subjects

	Controls	NTDs^a^	*P*
Number of foetus for miRNA Array	5	5	
Gender (%)^b^			
Male	2 (40.0)	1 (20.0)	0.49
Female	3 (60.0)	4 (80.0)	
Gestational week^c^ Median (Min~Max)	21 (20~22)	21 (20~22)	0.74
Number of foetus for has‐let‐7 g	50	57	
Gender (%)^b^			
Male	22 (44.0)	24 (42.1)	0.783
Female	28 (56.0)	33 (57.9)	
Gestational week (%)^c^			
≤16	5 (10.0)	4 (7.0)	0.04
17–24	40 (80.0)	35 (61.4)	
≥25	5 (10.0)	18 (31.6)	
Foetal brain folate (ng/mg)^d^	0.133 ± 0.039	0.102 ± 0.032	0.01

a NTDs used here are spinal bifida.

b Chi‐squared test was used to calculate the *P‐*values.

c *t*‐test was used to calculate the *P‐*values.

d number tested for folate: 23 controls and 39 NTDs.

The methylation levels of 22 miRNA promoters were assayed in five NTD cases and compared with five controls, the data for which are shown in Table 2. Among them, eight miRNAs, namely hsa‐let‐7 g, hsa‐mir‐193b, hsa‐mir‐210, hsa‐mir‐218‐2, hsa‐mir‐30e, hsa‐mir‐32, hsa‐mir‐34b and hsa‐mir‐7‐1, showed significant methylation differences between these two groups. All of these eight miRNAs lost their methylation modifications in NTDs, with these losses ranging from 0.42% to 14.21%. With the exception of hsa‐let‐7 g, the other seven miRNAs shared relatively similar hypomethylation levels in both controls and NTDs (methylation level ≤10%), which means weaker roles in methylation regulation.

**Table 2:** Methylation level of 22 miRNAs in promoter regions in NTDs and controls

miRNA Gene ID	Controls (Mean ± S.D., %) *n* = 5	NTDs (Mean ± S.D., %) *n* = 5	*P*
hsa‐let‐7 g	**25.46 ± 4.72**	**11.34 ± 5.35**	**0.00**
hsa‐let‐7i	0.02 ± 0.02	0.05 ± 0.03	0.11
hsa‐mir‐10a	3.77 ± 3.10	1.31 ± 0.89	0.13
hsa‐mir‐1‐1	36.76 ± 14.33	27.22 ± 32.68	0.62
hsa‐mir‐124‐2	13.18 ± 6.01	12.82 ± 4.74	0.92
hsa‐mir‐126	17.93 ± 16.56	25.58 ± 41.56	0.74
hsa‐mir‐149	0.02 ± 0.04	0.04 ± 0.03	0.43
hsa‐mir‐155	0.37 ± 0.15	0.24 ± 0.09	0.15
hsa‐mir‐15b	0.21 ± 0.20	0.49 ± 0.54	0.31
hsa‐mir‐17	0.11 ± 0.08	0.14 ± 0.08	0.48
hsa‐mir‐191	0.10 ± 0.13	0.17 ± 0.13	0.44
hsa‐mir‐193b	**3.46 ± 0.90**	**1.13 ± 0.76**	**0.00**
hsa‐mir‐210	**6.59 ± 1.72**	**2.33 ± 1.21**	**0.00**
hsa‐mir‐218‐1	0.10 ± 0.10	0.41 ± 0.43	0.15
hsa‐mir‐218‐2	**0.83 ± 0.23**	**0.41 ± 0.09**	**0.00**
hsa‐mir‐24‐1	99.85 ± 0.06	99.89 ± 0.10	0.50
hsa‐mir‐301a	1.81 ± 0.41	3.18 ± 1.78	0.13
hsa‐mir‐30e	**4.23 ± 1.55**	**1.76 ± 0.49**	**0.01**
hsa‐mir‐32	**6.69 ± 2.43**	**2.78 ± 1.33**	**0.01**
hsa‐mir‐34b	**16.87 ± 5.47**	**6.06 ± 4.82**	**0.02**
hsa‐mir‐378a	0.40 ± 0.15	11.33 ± 24.67	0.41
hsa‐mir‐7‐1	**2.26 ± 0.53**	**1.08 ± 0.44**	**0.00**

Differences between the NTD and control groups were analysed by Student's *t*‐test. Bold values: p<0.05.

**Figure 2:**
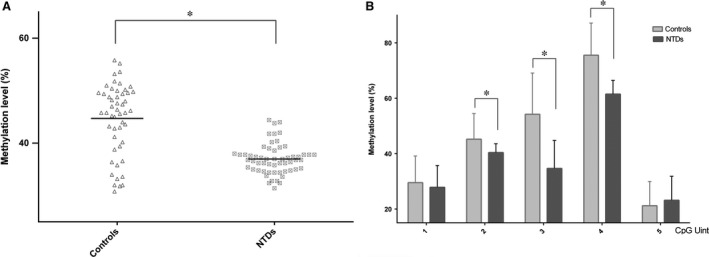
Methylation levels of hsa‐let‐7 g in NTD cases and controls. (**A**) Mean methylation levels of hsa‐let‐7 g in NTD cases and controls. (**B**) Methylation levels at all CpG sites in hsa‐let‐7 g between samples from subjects. CpG sites are numbered 1–5 from the 5′ end to the 3′ end in the promoter area of hsa‐let‐7 g. *P < 0.05 by Student's *t*‐test. control: *n* = 50, NTD cases: *n* = 57.

The promoter region of hsa‐let‐7 g was selected and measured in nervous tissue from both NTD and control samples using the EpiTyper platform. Using two pairs of primers designed to amplify chr3: 52302917–52303573, five CpG units were assayed in 57 NTD and 50 control samples after bisulphite conversion of genomic DNA. The mean methylation level of hsa‐let‐7 g in the NTD samples was significantly lower than that in the control samples (36.98 ± 0.38% compared with 44.70 ± 0.95%, *P* < 0.001, Fig. 2A). Our data show a striking reduction in the methylation level of hsa‐let‐7 g in the NTD group; the methylation level in all NTD cases was lower than the mean level in the control group. The methylation levels of every CpG unit within hsa‐let‐7 g were also evaluated. As shown in Figure 2B, methylation levels varied among the different CpG units; taken together, compared with the control samples, the methylation levels of the middle three CpG units in NTDs were significantly lower (*P* ≤ 0.001), whereas no significant differences were found in the first and fifth CpG units.

In an attempt to develop a model for assessing the risk of developing NTDs based on the methylation level of hsa‐let‐7 g, NTD samples were categorized according to the quartiles of methylation level found in the control samples. Based on the hsa‐let‐7 g methylation level, 94.73% of NTD samples were grouped into the lowest quartile (methylation level <43.01%). Only three NTD samples (5.26%) were grouped into the highest quartile (methylation level ≥43.01%). As shown in Table 3, low methylation levels increased the risk of NTDs 42‐fold (OR: 42.00; 95% CI: 15.28–115.44) compared with high methylation levels. After adjusting the OR to take into account gestation time and sex, there was still a risk of NTDs associated with low hsa‐let‐7 g methylation levels (AOR: 38.02; 95% CI: 28.88–50.12).

**Table 3:** Risk of NTDs associated with methylation levels of hsa‐let‐7 g

Methylation level	NTDs *n* (%)	Control *n* (%)	OR (95% CI)	*P*	Adjusted OR^a^ (95% CI)	*P*
	*n* = 57	*n* = 50				
>*P*_25_	3 (5.26)	35 (70.00)	1 (reference)		1 (reference)	
≤*P*_25_	54 (94.73)	15 (30.00)	42.00 (15.28–115.44)	0.001	38.02 (28.88–50.12)	0.001

NTDs, neural tube defects; OR: odds ratio; CI: confidence interval.

Cut‐off value is defined as the 25th percentile of methylation level in the control group, *P*_25_: 25th percentile.

a Adjusted for sex and gestation week by logistic regression.

**Figure 3:**
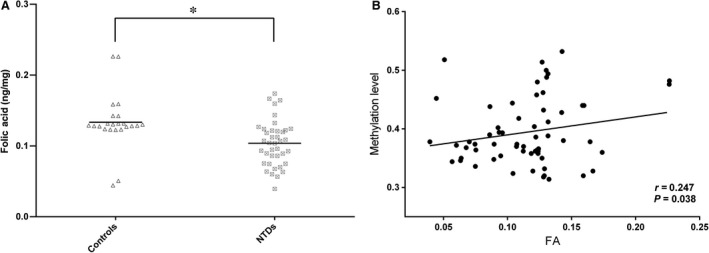
Relationship between folate metabolism and hsa‐let‐7 g methylation level. (**A**) Folic acid levels in brain tissue in NTD cases and controls. Cases: *n* = 44, controls: *n* = 23. *P < 0.01. (**B**) Correlation between folate levels in brain tissue and methylation status of hsa‐let‐7 g. The abscissa represents the concentration of folate in brain tissue. The ordinate represents the ratio of methylation of each sample. The folate level had a significant positive correlation with the hsa‐let‐7 g methylation level, *r* = 0.247, *P* < 0.05.

As folic acid metabolism is believed to be connected to methylation status, we measured the concentrations of folic acid in foetal brain tissues to investigate the possible associations between one‐carbon metabolism and the regulation of hsa‐let‐7 g methylation. As shown in Figure 3, folic acid metabolism was disturbed in the foetuses; compared with the control samples, patient brain tissue samples had lower folic acid concentrations than controls (0.102 ± 0.032 ng/mg compared with 0.133 ± 0.039 ng/mg, *P* < 0.01, Fig. 3A). We further analysed the underlying relationship between the methylation level and folic acid level, as shown in Figure 3B; a significant positive correlation was found between hsa‐let‐7 g methylation level and folic acid concentration in brain tissues (*r* = 0.246, *P* < 0.05). Our data imply that the methylation level of hsa‐let‐7 g is regulated by folic acid metabolism.

**Figure 4:**
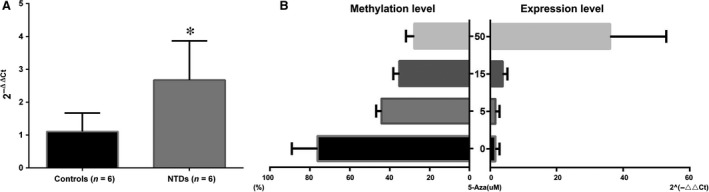
Increased expression level of hsa‐let‐7 g accompanied by hypomethylation in NTDs and HCT15 cells treated with 5‐Aza. (**A**) ORF1p expression levels were compared between NTDs and controls. Increased expression of hsa‐let‐7 g was detected in samples from brain tissues in NTD cases (*r* = 2.42, *P* < 0.05). (**B**) Correlation between hsa‐let‐7 g expression level and hsa‐let‐7 g hypomethylation in HCT‐15 cells. Cells were treated with 0, 5, 15 or 50 μM 5‐Aza and assayed for hsa‐let‐7 g expression using qPCR and levels of hsa‐let‐7 g methylation.

To evaluate whether hsa‐let‐7 g hypomethylation in cases of NTDs increased its own expression level, this level was measured in brain tissue samples using real‐time PCR for six NTD cases and six controls. As expected, the expression level of hsa‐let‐7 g in the NTD cases was more than double higher compared with that in the controls (*r* = 2.42, *P* < 0.05). Next, we explored whether the hsa‐let‐7 g expression level was affected by promoter methylation; this potential association was assayed in HCT15 cells treated with 5‐Aza. As shown in Figure 4B, hsa‐let‐7 g methylation levels decreased in a dose‐dependent manner in the different groups of HCT‐15 cells treated with 5‐Aza (at 5, 15 and 50 μM/μl). Accordingly, the transcription of gli2 was significantly increased in the treated groups compared with that in the untreated group. In particular, the expression level of hsa‐let‐7 g in the group of cells treated with the highest concentration of 5‐Aza (50 μM/μl) was significantly higher than that of cells in the groups treated with lower concentrations (5 and 15 μM/μl). This implies that hsa‐let‐7 g level is sensitive to changes in promoter methylation.

**Figure 5:**
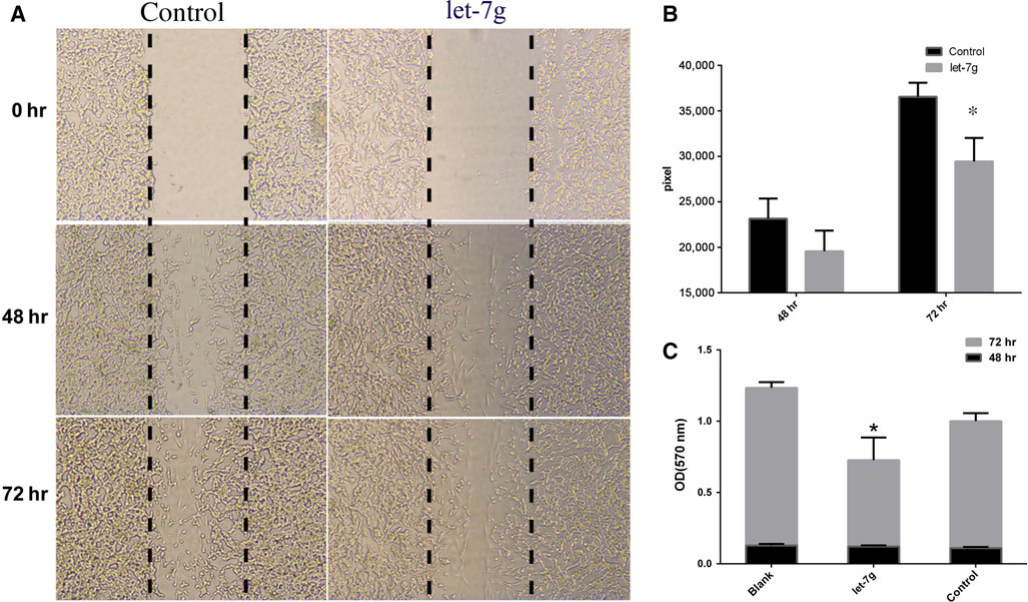
Effects of let‐7 g on cell migration and proliferation. (**A)** Status of cell migration 0, 48 and 72 hrs after let‐7 g transfection. (**B**) Migration assay indicated that let‐7 g overexpression inhibited SK‐N‐SH cell migration at 72 hrs. *P < 0.05, compared with the control group. (**C**) The proliferation of SK‐N‐SH cells was determined after transfection with let‐7 g at different time‐points; cell proliferation decreased at 72 hrs after let‐7 g transfection. *P < 0.05, compared with the control group.

Further analyses were performed to determine the functional roles of hsa‐let‐7 g. For example, we determined its role in tumour cell invasion. Specifically, we performed cell migration analysis, which revealed that the overexpression of hsa‐let‐7 g led to more rapid invasion than that of vector‐transfected SK‐N‐SH cells (Fig. 5A and B). MTT assay was also performed to assess the effect of hsa‐let‐7 g on cell proliferation. Our data confirmed that enforced overexpression of hsa‐let‐7 g in SK‐N‐SH cells resulted in a higher proliferation rate than in the control group at 72 hrs after hsa‐let‐7 g transfection (Fig. 5C).

To elucidate the mechanisms by which hsa‐let‐7 g acts in cases of NTDs associated with folate deficiency, we predicted its potential target genes using the miRWalk database. As listed in Table S2, thousands of genes were suggested as possible targets of hsa‐let‐7 g. The results of genomewide profiles of human gene expression comparing two NTD cases and controls were used to select potential hsa‐let‐7 g target genes involved in folate‐related NTDs. Genes with an increase in expression of >twofold or a decrease to <0.5‐fold in both of the pair of NTD samples were selected and compared with the hsa‐let‐7 g potential target genes. Among these candidates, 13 genes (including nine up‐regulated and four down‐regulated genes) were predicted as target genes in miRWalk and were selected for further analysis (Table 4).

**Table 4:** Changes in human gene expression between NTD cases and controls

Gene name	Fold change (NTD1 *versus* Controls) n_control_ = 3	Fold change (NTD2 *versus* Controlsl) n_control_ = 3	Regulation
CCND1	6.3826413	9.471867	up
COL1A1	70.449165	17.862146	up
SMOX	2.1050968	12.563275	up
ADM	4.911256	17.1695	up
EGR1	7.3365874	11.726604	up
CDKN1A	3.1844065	37.75585	up
DCN	19.248352	6.3234243	up
IL1B	7.0923753	4.698134	up
SCPEP1	6.028733	4.552225	up
DNMT3B	2.1543741	2.1252298	down
FBXW7	2.2386136	3.9801338	down
PGM2L1	2.0143974	4.4398966	down
VSNL1	2.254502	3.051205	down

**Figure 6:**
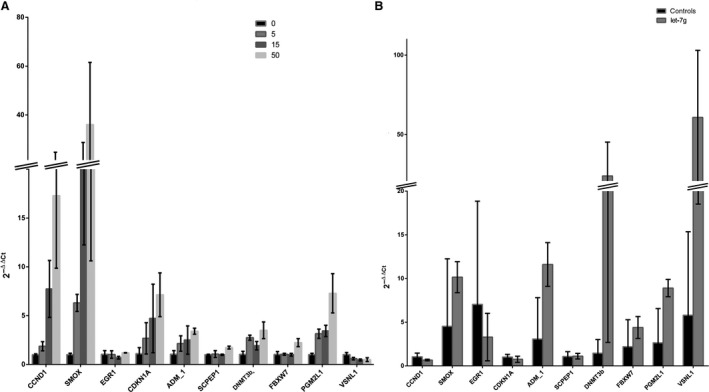
mRNA levels of predicted target genes were measured by RT‐qPCR. (**A**) mRNA expression levels in HCT‐15 cells treated with 5‐Aza. Among them, the expression of CCND1, SMOX, ADM_1, CDKN1A, DNMT3B and PGM2L1 genes increased significantly, while that of the VSNL1 gene decreased significantly, with the loss of hsa‐let‐7 g methylation. (**B**) mRNA expression levels in SK‐N‐SH cells transfected with let‐7 g. Among the genes, the expression of SMOX, ADM_1, DNMT3B, PGM2L1 and VSNL1 increased significantly with a high level of let‐7 g.

All of these candidate genes were further examined in cell lines with hsa‐let‐7 g methylation gradation and hsa‐let‐7 g‐overexpressing cells to determine whether hsa‐let‐7 g could regulate target gene expression. This was indeed the case for 10 of the 13 genes, the results for which are shown in Figure 6. With the loss of methylation of hsa‐let‐7 g, there were increases in the expression of the CCND1, SMOX, ADM and CDKN1A genes, and a decrease in that of the VSNL1 gene, which are in accordance with the results in the NTD cases; in contrast, EGR1, SCPEP, DNMT3B, FBXW7 and PGMIL1 genes showed contradictory results compared with the NTD samples. On the other hand, in hsa‐let‐7 g‐overexpressing cell lines, only the EGR1 and SMOX genes showed changes in their expression similar to those in NTD cases; the expression of 11 other genes changed differently upon hsa‐let‐7 g overexpression from that in NTD cases. Taking these findings together, only the SMOX gene was identified as a potential target gene regulated by hsa‐let‐7 g level and hsa‐let‐7 g hypomethylation. In summary, these findings suggest that hsa‐let‐7 g may exert its biological function in NTD cases associated with folate deficiency by regulating the expression of its target genes, such as SMOX.

## Discussion

This study identified the connection between aberrant folic acid metabolism and miRNA regulation through alterations in DNA methylation in the pathogenesis of NTDs. A change in the level of hsa‐let‐7 g, which has been proved to be regulated by promoter methylation, was suggested to participate in the development of NTDs associated with folate deficiency. Specifically, hypomethylation of hsa‐let‐7 g increased the risk of NTDs, which may shed some light on the mechanisms involved in the development of these defects.

miRNAs have been shown to play critical roles in human brain development; for example, the maintenance and regulation of endogenous miRNA levels are critical during mammalian brain development 31. Previous studies also suggested that miRNA alterations occur during neural stem cell differentiation and morphological development of the mammalian brain 29, 32. One member of our group also proved that a significant difference in miRNA expression pattern is a possible marker of anencephaly 17. Although it has been proved that miRNA expression is dysregulated in NTD cases, the molecular mechanism underlying this has not been understood.

It has suggested that insufficient folate intake is a cause of NTDs 33. Impaired DNA methylation is believed to participate in this process 16, 34. Numerous investigations have indeed indicated that the aberrant methylation of miRNA around the promoter regions contributes to dysregulation of its expression 35. Our work is aimed at clarifying whether modification of the methylation level participates in the regulation of folic acid and miRNAs in cases of NTDs. In this study, a total of 22 miRNAs were investigated for changes in methylation at their promoter; eight showed significant changes of this type in NTD cases. All of these changes implied that more or less biological function changes caused by methylation regulations in NTDs with miRNA involved. However, given the limitation of MassArray Platform, which could only accurately detect a range of methylation level from 5% to 95%, only hsa‐let‐7 g was selected for further investigation in this study.

In the case of hsa‐let‐7 g, we identified intense hypomethylation in brain tissues of NTD cases, which correlated with silencing of its expression. Using HCT15 cell line, which is an extensive methylation model suitable for studying biological function aberrations caused by methylation decreasing, we found that hsa‐let‐7 g methylation and expression level could also be regulated by the demethylating agent 5‐aza‐2‐deoxycytidine, indicating that DNA methylation is a major mechanism for inhibiting hsa‐let‐7 g expression. The relationship between hypomethylation of hsa‐let‐7 g and folic acid concentration in NTDs provides indirect evidence that, in the pathogenesis of NTDs, folate deficiency may disturb hsa‐let‐7 g through DNA methylation. The methylation level of hsa‐let‐7 g in all NTD cases was lower than half the level in controls, implying that it might play a novel and important role as a biomarker of NTDs.

The let‐7 family is composed of 12 members (let‐7‐a1, a2, a3, b, c, d, e, f1, f2, g, i, and miR‐98) located on eight different chromosomes 36. It has been demonstrated that let‐7 family members promote cell proliferation 37, 38 and are associated with hypertension and atherosclerosis 39, 40. Among the members of this family, hsa‐let‐7 g was reported to be involved in migration and proliferation 41, 42, 43. Our data also confirmed that the overexpression of hsa‐let‐7 g disturbed the migration and proliferation of neuroblastoma cells. Changes in miRNA transcripts may exert effects through hundreds of target genes. Therefore, to further elucidate the target genes of hsa‐let‐7 g and the mechanisms involved in NTDs, we performed a combined analysis of the results of genomic expression microarray of NTDs and bioinformatic prediction using miRWalk; from this, we found that nine and four genes were up‐regulated and down‐regulated in NTDs compared with controls. However, only the SMOX gene was validated in hsa‐let‐7 g demethylation cells and also in hsa‐let‐7 g‐overexpressing cells. Further studies on the roles of hsa‐let‐7 g in NTDs through regulating SMOX gene expression may shed new light on the mechanism behind the development of NTDs.

Our study is subject to some limitations. For the conduction limited of human sample collection, it is difficult to obtain brain tissue RNAs with high quality, expression data of target genes were based on only 2 *versus* 3 human samples; meanwhile, qualities of RNA were controlled by absorbance detection and gel electrophoresis, imperfections in these limited the reliability of conclusion in human study and further experiments in a larger sample size will increase robust estimate of mechanism of has‐let‐7 g regulation in the pathology of NTDs.

## Conclusion

In summary, our data provide evidence supporting the relationship between aberrant methylation of hsa‐let‐7 g and disturbed folate metabolism in the emergence of NTDs, implying that improvements in nutrition during early pregnancy may prevent NTDs, possibly *via* the donation of methyl groups for miRNAs. The miRNA hsa‐let‐7 g, along with its target genes, plays important roles in the prevention of NTDs by folic acid.

## Disclosure

The authors declare they have no competing interests as defined by Molecular Medicine, or other interests that might be perceived to influence the results and discussion reported in this paper.

## Supporting information


**Table S1** List of primers used in this study.

**Table S2** MicroRNA Target Results.
