# Supplementary material for: Folate deficiency disturbs hsa‐let‐7 g level through methylation regulation in neural tube defects
Source: J Cell Mol Med. 2017 Jun 19;21(12):3244–53. doi: 10.1111/jcmm.13228 (PMC5706510; doi:10.1111/jcmm.13228)
Supplement: Supplementary file 1 — Table S1 List of primers used in this study. [file JCMM-21-3244-s001.doc]

Table S1 List of primers used in this study

| **Gene Name** | **Primer** |
| --- | --- |
| hsa-let-7g | UGAGGUAGUAGUUUGUACAGUU |
| mmu-let-7g-5p | UGAGGUAGUAGUUUGUACAGUU |
| CCND1_L | CTACACCGACAACTCCAT |
| CCND1_R | GATGATCTGTTTGTTCTCCTC |
| SMOX_L | GTAACCGCATCAGGAATG |
| SMOX_R | AGGTACTGCTGGATCATG |
| ADM_L | CCAGAGCATGAACAACTTC |
| ADM_R | CTTGTCCTTATCTGTGAACTG |
| EGR1_L | TCTGAACAACGAGAAGGT |
| EGR1_R | ACTGACCAAGCTGAAGAG |
| CDKN1A_L | AGACCAGCATGACAGATT |
| CDKN1A_R | ACTAAGGCAGAAGATGTAGAG |
| SCPEP1_L | AAGCCAGTCATTAGCATTG |
| SCPEP1_R | TCCAAAGATTTAGGGTCACT |
| FBXW7_L | GACGCCGAATTACATCTG |
| FBXW7_R | ACTGGGGTTCTATCACTTG |
| VSNL1_L | CTCAAGCAGTGGTACAAAG |
| VSNL1_R | CTCTCGGAAGTCAATGGT |
| DNMT3B_L | GATGACGATGGCTATCAGT |
| DNMT3B_R | CGGGAGACACATGTAACA |
| PGM2L1_L | AGAATGTGTGGAACCCTG |
| PGM2L1_R | CAAGGTGGTCTTCGAGTT |
